# Sex differences in the impact of arterial stiffness on cerebrovascular function

**DOI:** 10.1002/alz.092998

**Published:** 2025-01-03

**Authors:** Emily H Reeve, Ainsley P Hogan, Abigail E Cullen, Ashley E Walker

**Affiliations:** ^1^ University of Oregon, Eugene, OR USA

## Abstract

**Background:**

Stiffening of the large arteries is a hallmark feature of vascular aging and is associated with cognitive impairment and Alzheimer’s disease pathology. Increased large artery stiffness leads to higher‐than‐normal pulse pressure in the cerebral circulation, damaging endothelial cells. It is known that short‐term exposure to stiffer large arteries causes cerebral artery endothelial dysfunction and hypoperfusion in young mice. However, the impact of long‐term exposure to large artery stiffness on cerebrovascular function is unknown. As there are known sex differences in Alzheimer’s disease risk, we sought to understand the influence of sex on the impact of large artery stiffness on cerebrovascular function.

**Method:**

We studied an established model of greater large artery stiffness, the elastin haploinsufficient (Eln±) mouse, at young (6 months) and old (24 months) ages and compared with wildtype (Eln+/+) mice. We measured endothelium‐dependent dilation ex vivo in pre‐constricted, pressurized posterior cerebral arteries by the maximal response to acetylcholine. Nitric oxide bioavailability was determined by the acetylcholine response with and without the presence of L‐NAME, a nitric oxide synthase inhibitor. Endothelium‐independent dilation was assessed by the maximal response to sodium nitroprusside.

**Result:**

With the sexes combined, posterior cerebral artery endothelium‐dependent dilation was better for young compared with old mice (p<0.001) and for Eln+/+ compared with Eln± mice (p = 0.03). In males, Eln+/+ mice had better posterior cerebral artery endothelium‐dependent dilation than Eln± mice at young ages (p = 0.05), but not at old ages (p = 0.35). The opposite occurred in females, as Eln+/+ mice had better posterior cerebral artery endothelium‐dependent dilation than Eln± at old ages (p = 0.04) but not at young ages (p = 0.71). Group differences in endothelium‐dependent dilation were mediated by differences in nitric oxide bioavailability. There was no effect of genotype, age, or sex on endothelium‐independent dilation.

**Conclusion:**

Our results suggest males are more vulnerable at young ages, while females are more vulnerable at old ages, to the detrimental effects of large artery stiffness on cerebrovascular function. These age‐dependent differences in vulnerability could underlie sex differences in Alzheimer’s disease risk. Funded by AARG‐200675709, TL1TR002371.

